# Van der Waals epitaxial growth and optoelectronics of a vertical MoS_2_/WSe_2_ p–n junction

**DOI:** 10.1007/s12200-022-00041-4

**Published:** 2022-10-11

**Authors:** Yu Xiao, Junyu Qu, Ziyu Luo, Ying Chen, Xin Yang, Danliang Zhang, Honglai Li, Biyuan Zheng, Jiali Yi, Rong Wu, Wenxia You, Bo Liu, Shula Chen, Anlian Pan

**Affiliations:** 1 Key Laboratory for Micro-Nano Physics and Technology of Hunan Province, College of Materials Science and Engineering, Hunan University, Changsha, 410082 China; 2 School of Materials Science and Engineering, Key Laboratory for Micro-Nano Physics and Technology of Hunan Province, Hunan University, Changsha, 410082 China

**Keywords:** MoS_2_, WSe_2_, Chemical vapor deposition (CVD), Vertical heterostructure, Optoelectronic transistor

## Abstract

Two-dimensional (2D) transition metal dichalcogenides (TMDs) have attracted extensive attention due to their unique electronic and optical properties. In particular, TMDs can be flexibly combined to form diverse vertical van der Waals (vdWs) heterostructures without the limitation of lattice matching, which creates vast opportunities for fundamental investigation of novel optoelectronic applications. Here, we report an atomically thin vertical p–n junction WSe_2_/MoS_2_ produced by a chemical vapor deposition method. Transmission electron microscopy and steady-state photoluminescence experiments reveal its high quality and excellent optical properties. Back gate field effect transistor (FET) constructed using this p–n junction exhibits bipolar behaviors and a mobility of 9 cm^2^/(V·s). In addition, the photodetector based on MoS_2_/WSe_2_ heterostructures displays outstanding optoelectronic properties (*R* = 8 A/W, D* = 2.93 × 10^11^ Jones, on/off ratio of 10^4^), which benefited from the built-in electric field across the interface. The direct growth of TMDs p–n vertical heterostructures may offer a novel platform for future optoelectronic applications.

**Graphical Abstract:**

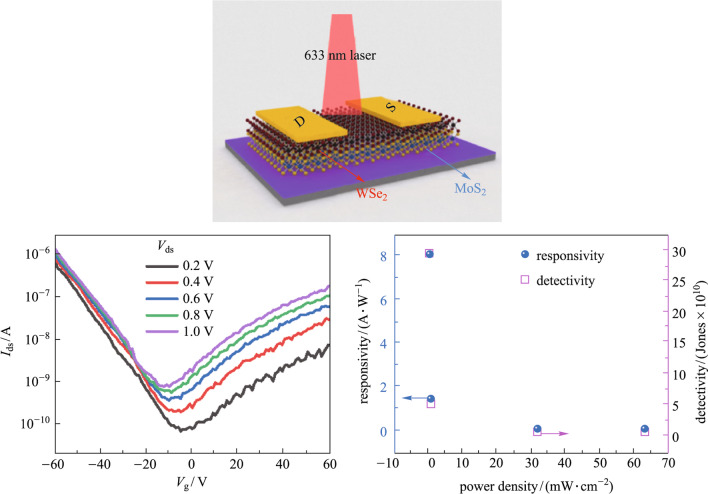

**Supplementary Information:**

The online version contains supplementary material available at 10.1007/s12200-022-00041-4.

## Introduction

In the past decades, two-dimensional (2D) transition metal dichalcogenides (TMDs) layered semiconductors have been considered as promising candidates for next-generation electronics and optoelectronics. This is due to their unique properties such as atomically thin thickness, and to their electronic, photonic, and mechanical properties [1–6]. Moreover, the heterostructures that are composed of various 2D layered TMDs materials have attracted more and more attention as a consequence of their diversified band alignments [5]. For example, type-I materials are usually used for light-emitting diodes [7], type-II have an important application in photovoltaic devices [8, 9], and type-III are widely used in the tunneling field effect transistors [10]. Until now, there have been diverse ways to synthesize the heterostructures, including mechanical stacking, the solution method, and chemical vapor deposition (CVD) [12–14], in which the CVD approach can easily synthesize materials with high production yield and large area. Many van der Waals heterostructures have been successfully composed by the CVD method and can be used in integrated optoelectronic applications [15–17]. For instance, Li et al. reported high-performance optoelectronic devices based on van der Waals vertical MoS_2_/MoSe_2_ heterostructures with enhanced photoresponsivity of 36 A/W and a remarkable detectivity of 4.8 × 10^11^ Jones [16]. Wu et al. demonstrated the vapor growth of WSe_2_/WS_2_ heterostructures with distinct nonlinear optical properties by controlling crystallographic alignments in the form of AA and AB stacking [15]. However, although there is demand for mass production of high-quality vertical p–n heterostructures for use in integrated devices, there are still few reports on them because synthesizing the non-alloyed heterojunctions with sharp interface by CVD method is difficult. Most of the devices developed so far are based on single-material [18–21], but their performance is limited by defects and small light absorption cross-sections [21, 22]. Therefore, the controllable synthesis of vertical p–n heterostructures is very important for future integrated electronic and optoelectronic applications.

In this work, we report production of vertical MoS_2_/WSe_2_ p–n heterojunctions through a two-step CVD method and explore its application in photoelectric devices. The optical measurements and scanning transmission electron microscopy (STEM) demonstrate that the heterostructures are of high quality, without alloying. Electrical measurements exhibit considerable photon-electron conversion efficiency, photoresponsivity, detectivity, and stable light response. The study thus provides an alternative approach to design various TMDs heterostructures for future optoelectronic applications.

## Experimental details

### Synthesis of MoS_2_/WSe_2_ vertical heterostructures

The vertical MoS_2_/WSe_2_ heterostructures were synthesized by a two-step growth CVD approach. In the first step, MoS_2_ template was synthetized using a salt-assisted method, Salt could reduce the evaporation temperature of MoO_3_, thereby reducing the difficulties for production of MoS_2_ with large-area. An alumina boat containing S powder was placed at upstream of a furnace. Then, another boat with mixed powder of NaCl and MoO_3_ (99.99%, Alfa Aesar) was positioned at the central region of furnace, on top of which three pieces of SiO_2_/Si substrates was placed for deposition. Before heating, the system was cleaned by pure Ar flow for 30 min at a rate of 80 standard cubic centimeters per minute (sccm). The furnace was heated to 810 ℃ within 30 min at 60 sccm, with Ar as the carrier gas, then maintained at that temperature for a further 10 min. Finally, the furnace naturally cooled down to room temperature. For the second step, to synthesize vertical WSe_2_/MoS_2_ heterostructures, a boat with as-grown MoS_2_ templates was placed at downstream of furnace for subsequent deposition of WSe_2_. The WSe_2_ source powder (99.99%, Alfa Aesar) was put at the central heating area of the furnace. After the cleaning procedure as described above, the furnace was heated to 1020 ℃ within 30 min at 60 sccm and maintained at that temperature for 3 min. After the reaction, the furnace cooled down to room temperature naturally.

### Characterizations

The morphologies of heterostructures were characterized using an optical microscopy (Zeiss Axio Scope A1), an atomic force microscope (AFM, Bruker Multimode 8), and a scanning transmission electron microscope (JEM ARM200F). The optical measurements (Photoluminescence and Raman) were conducted using a confocal microscope (WITec, alpha-300) with a 532 nm laser focused by an objective.

### Device fabrication and optoelectronic measurements

Standard e-beam lithography (EBL, Raith 150) and metal thermal evaporation were performed to fabricate the Au/Cr (10 nm/50 nm) electrodes on the as-grown heterostructures with a lift-off approach. The electric and optoelectronic properties of the heterostructures were measured in vacuum with Lake Shore Probe Station and Agilent B1500A semiconductor analyzer at room temperature.

## Results and discussion

**Fig. 1:**
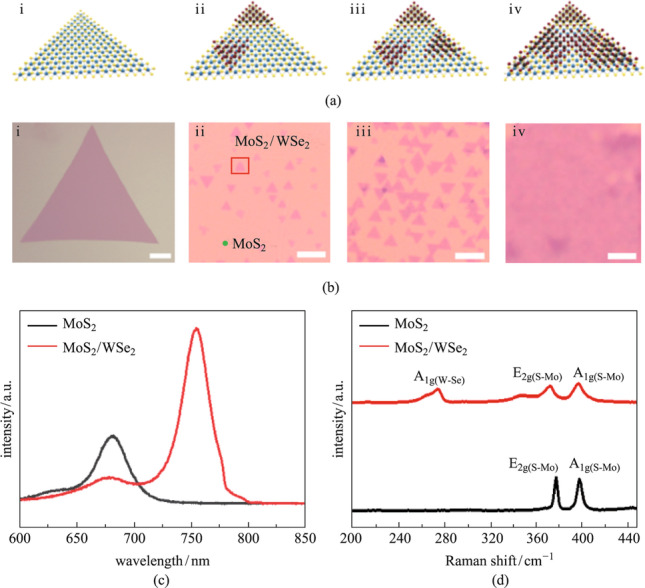
Growth morphology and the optical characterizations of as-grown MoS_2_/WSe_2_ heterostructures. Schematic views of heterostructure growth (**a**_i_–**a**_iv_) and their corresponding optical images (**b**_i_–**b**_iv_). Scale bar: 20 µm. **c** PL spectra of monolayer MoS_2_ and MoS_2_/WSe_2_ heterostructure. **d** Raman spectra of MoS_2_ and MoS_2_/WSe_2_ heterostructure

The MoS_2_/WSe_2_ heterostructures were grown by a two-step CVD method. Figure 1a shows a schematic diagram of the atomic growth model, and Fig. 1b exhibits a picture of the heterostructure under the optical microscope. The schematic view and optical image of MoS_2_ grown on SiO_2_/Si substrate are shown in Figs. 1a_i_ and 1b_i_ (Additional file 1: Fig. S1 for detail) respectively. The lateral size of monolayer MoS_2_ triangles ranged from 20 to 200 μm. The red square in Fig. 1b_ii_ indicates the small WSe_2_ triangles that was grown on MoS_2_ in the second step, and the area marked by the green spots is the underlying MoS_2_. The evolution of morphology of WSe_2_ on MoS_2_ after 2, 4, and 6 min growth is shown in Figs. 1a_ii_–a_iv_ and 1b_ii_–b_iv_, respectively, with the whole WSe_2_ covered eventually. The optical image clearly indicated that WSe_2_ on MoS_2_ had only 0° and 60° stacking modes. Atomic force microscopy showed that MoS_2_ and WSe_2_ were both monolayers (Additional file 1: Fig. S2). Figure 1c shows the photoluminescence (PL) spectra of MoS_2_ and MoS_2_/WSe_2_ heterostructures. The peaks at 680 and 755 nm correspond to the A excitons of MoS_2_ and WSe_2_, respectively. The PL intensity of MoS_2_ becomes quenched in the heterostructure because of the charge transfer in the MoS_2_/WSe_2_ interface, with type-II band alignment, as shown in Fig. 4f. Figure 1d shows the Raman spectra of MoS_2_ and MoS_2_/WSe_2_heterostructure. The peak positions of MoS_2_ locate at 379 cm^−1^ (E_2g(S-Mo)_) and 397 cm^−1^ (A_1g(S-Mo)_), respectively, while that of the heterostructure locate at 270 cm^−1^ (A_1g(W-Se)_), 372 cm^−1^ (E_2g(S-Mo)_), and 400 cm^−1^ (A_1g(S-Mo)_). PL and Raman results are consistent with MoS_2_ and WSe_2_ properties previously reported, proving the successful synthesis of MoS_2_/WSe_2_ heterostructure.

**Fig. 2:**
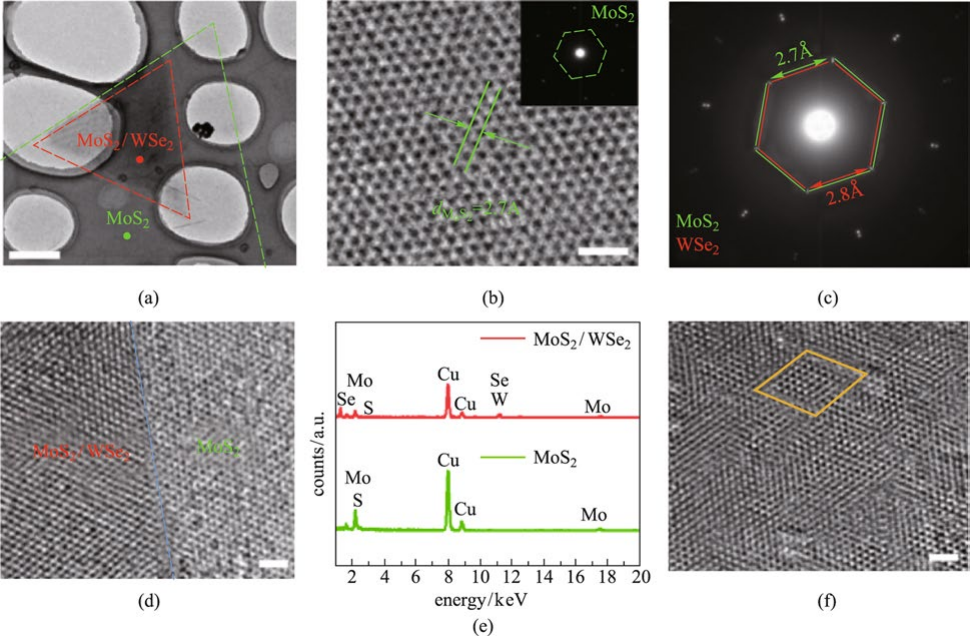
High-resolution atomic characterizations of MoS_2_/WSe_2_ heterostructure. **a** Low-resolution TEM image of transferred MoS_2_/WSe_2_ heterostructure on a Cu grid. Scale bar: 1 μm. **b** HRTEM image of MoS_2_. Scale bar: 1 nm. **c** SAED patterns of MoS_2_/WSe_2_ heterostructure. **d** Atomic resolution scanning TEM image of the heterostructure interface. Scale bar: 2 nm. **e** EDS spectra of the MoS_2_/WSe_2_ vertical heterostructure. **f** HRTEM image of heterostructure region. Scale bar: 2 nm

The MoS_2_/WSe_2_ heterostructure grown on SiO_2_/Si was transferred to a copper net (see the supporting information for the detail transfer method), and then the microstructure and chemical composition of the MoS_2_/WSe_2_ heterostructure were analyzed by transmission electron microscopy (TEM). Figure 2a gives the low-resolution scanning transmission electron microscope (TEM) image of MoS_2_/WSe_2_ vertical heterostructure. The triangular area marked by green dash line is the bottom layer of MoS_2_, and the red triangle is the heterojunction area. Figure 2b is the high-resolution transmission electron microscope (HRTEM) image of the bottom MoS_2_, with a lattice spacing of 2.7 Å, corresponding to the (100) crystal plane of the 2H phase MoS_2_. The inset is the selected area electron diffraction (SAED) pattern of MoS_2_. The SAED of the MoS_2_/WSe_2_ heterostructure is shown in Fig. 2c. Two sets of regular hexagon diffraction pattern are displayed clearly. The polygonal diffraction patterns marked by green and red lines have a crystal plane spacing of 2.7 Å and red 2.8 Å, corresponding to MoS_2_ and WSe_2_ (100) crystal planes, respectively. Figure 2d shows the area at the interface of the heterostructure under the HRTEM. The right side is MoS_2_, and the left side is the heterostructure. The atomic structure is clear, which proves that the high-quality MoS_2_/WSe_2_ vertical heterojunction with a clear interface was successfully synthesized. The TEM energy-dispersive X-ray photoelectron spectroscopy (EDS) of different regions is shown in Fig. 2e. The red line shows several peaks for elements such as W, Se, Mo, S, and Cu (from the copper mesh), corresponding to the MoS_2_/WSe_2_ heterojunction. The green line only shows Mo, S, and Cu peaks, corresponding to the MoS_2_. The results agrees with The X-ray photoelectron spectroscopy (XPS) measurement of the heterojunction given in Additional file 1: Fig. S3. Figure 2f is the HRTEM image in the heterojunction region, the moiré patterns can be clearly observed due to the interlayer coupling between MoS_2_ and WSe_2_. This observation further demonstrates the successful synthesis of the high-quality, well-defined MoS_2_/WSe_2_ heterostructure.

**Fig. 3:**
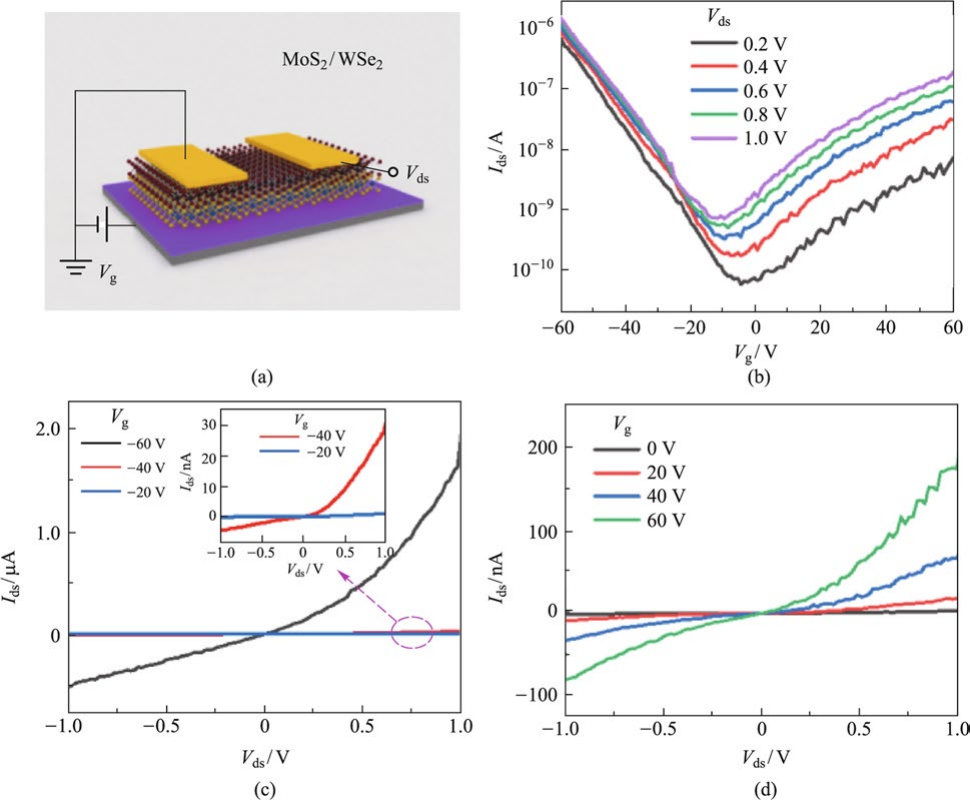
Electronic characterization of MoS_2_/WSe_2_ heterostructure transistors. **a** Schematic configuration. **b** Transfer characteristic curves. **c**, **d** *I*_ds_–*V*_ds_ output characteristics curves at various gate voltages ranging from − 60 to 60 V

To further investigate the charge transport properties of the vertically stacked MoS_2_/WSe_2_ van der Waals heterostructures, back-gate field-effect transistors (FET) were fabricated based on MoS_2_/WSe_2_ p–n heterojunctions. Figure 3a displays the device structure in which Ti/Au electrodes are deposited on WSe_2_, as the drain and source electrodes. The transport characteristic of the heterostructure device given in Fig. 3b shows ambipolar behavior, which is attributed to the p-type WSe_2_ and the n-type MoS_2_. Figure 3b shows that, when a negative gate voltage (*V*_g_) is applied, the WSe_2_ layer is turned on and the device exhibits p-type behavior. Conversely, when the MoS_2_ layer is on state and the device shows n-type behavior while the gate voltage was positive. At the same time, the current increases when the drain-source voltage *V*_ds_ changes from 0.2 to 1 V. The diagram indicates that the threshold voltage is − 10 V, and the on/off ratio is 10^4^. The mobility could be obtained using the expression *μ* = *g*_m_*L*/(*WC*_0_*V*_ds_), where *g*_m_ represents the transconductance, *L* = 17.3 μm and *W* = 54.5 μm are the length and the width of the device, respectively. *C*_0_ is the capacitance per unit area for the 280 nm thick SiO_2_. Based on the data shown in Fig. 3b, the calculated mobility is 9 cm^2^/(V·s). Figure 3c, d show the corresponding output characteristics of MoS_2_/WSe_2_ heterostructure. The inset in Fig. 3c is the enlarged view of the region marked by the red circle. The drain-source current *I*_ds_ decreases as the *V*_g_ varies from − 60 to − 20 V, and increases as *V*_g_ raises from 0 to 60 V. All the device measurements were conducted under vacuum at room temperature.

**Fig. 4:**
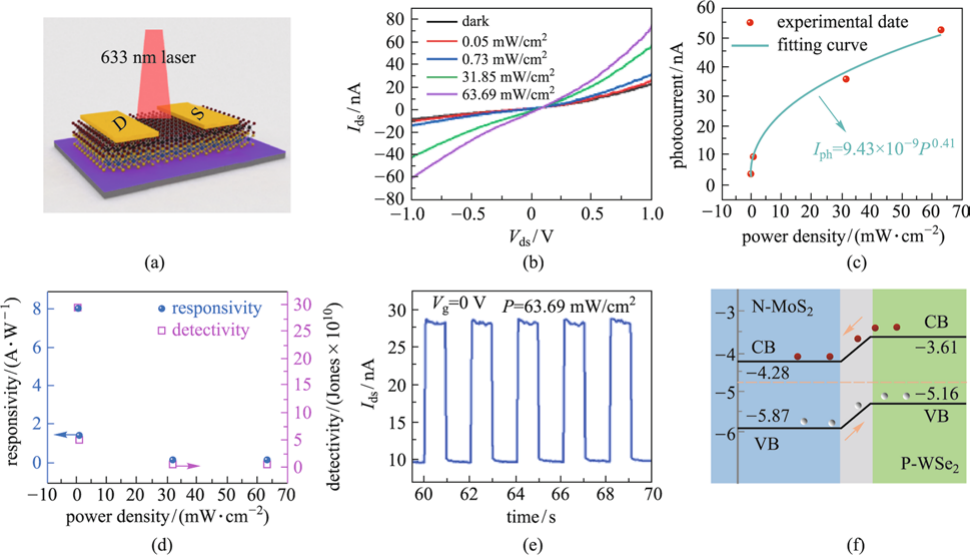
Optoelectronic device performance of MoS_2_/WSe_2_ vertical heterostructure. **a** Schematic illustration of the device. **b** *I*_ds_–*V*_ds_ curves across the heterojunction under 633 nm laser illumination with different incident powers. *V*_ds_ ranges from – 1 to 1 V. **c** Dependence of device photocurrent on illumination power densities. **d** Photoresponsivity and detectivity of the photodetector at various illumination power densities ranging from 0 to 63.69 mW/cm^2^. **e** Photocurrent response of the device; the laser excitation is turned on and off by a chopper working at 1 Hz (*V*_ds_ = 1 V, *V*_g_ = 0 V, *P* = 63.69 mW/cm^2^). **f** Band structures and the charge transfer process at the interface of the MoS_2_/WSe_2_ p–n heterojunction

Benefiting from the large built-in electric field at the p–n heterojunction interface and the resulting high separation efficiency of photogenerated carriers, p–n heterostructures are widely used in optoelectronic applications. Under illumination of 633 nm laser light, the photovoltaic and photoresponse characteristics of the MoS_2_/WSe_2_ vertical heterostrutures were examined further. The photodetector based on MoS_2_/WSe_2_ vertical heterjunction is schematically illustrated in Fig. 4a, with drain-source voltage *V*_ds_ applied to the heterojunction device. Figure 4b shows the *I*_ds_−*V*_ds_ curves at various illumination intensities ranging from 0 to 63.69 mW/cm^2^ (*V*_g_ = 20 V). More electron–hole pairs are generated as the illumination intensity increases, resulting in a greater photocurrent. In Fig. 4c, the photocurrent intensities are evaluated at a gate bias of 20 V. Other photocurrent data under different gate voltages are shown in Additional file 1: Fig. S4a. The photocurrent intensity (*I*_ph_) is defined as *I*_ph_ = *I*_light_ − *I*_dark_, where *I*_light_ (*I*_dark_) is current measured under light (dark state). The relation between *I*_ph_ and laser power density *P* can be expressed as *I*_ph_ = *aP*^*α*^. The fitted parameters of *a* and *α* obtained from Fig. 4c are 9.43 × 10^–9^ and 0.41, respectively. The responsivity (*R*) can be calculated using *R* = *I*_ph_/(*PA*), where *A* is the effective area of the device channel. The calculated responsivity of the device is up to 8 A/W (*P* = 0.05 mW/cm^2^, *V*_ds_ = 1 V, device area of 943.85 μm^2^), which is greater than that of monolayer MoS_2_ and WSe_2_ devices previously reported [24–26]. In addition, the value of *R* can reach up to 41 A/W at 60 V gate voltage (Additional file 1: Fig. S4b). To characterize the sensitivity of a photodetector, the device detectivity (D*) is used. D* can be calculated using the formula D* = *RA*^1/2^/ (2*eI*_dark_)^1/2^, where *e* is the electron charge, assuming D* is primarily affected by the shot noise of the dark current [26, 27]. According to this formula, D* can reach a maximum value of 2.93 × 10^11^ Jones as shown in Fig. 4d. Detectivity at various gate voltages can be found in Additional file 1: Fig. S4c. The photoresponse speed of the MoS_2_/MoSe_2_ heterostructure photodetector was investigated further by turning on and off the laser beam (633 nm, 63.69 mW/cm^2^). Figure 4e displays exemplary photocurrent curves for the photodetector, showing excellent stability and reliability of on/off switching behavior (*V*_ds_ = 1 V, *V*_g_ = 0 V, *P* = 63.69 mW/cm^2^). Figure 4f exhibits the band structure of the MoS_2_/WSe_2_. It is shown that a typical type-II heterostructure is formed. When the laser beam was focused on the heterojunction, the electrons in WSe_2_ were transferred to MoS_2_, whereas the holes in MoS_2_ were transferred to WSe_2_. This results in the photogating effect, wherein the charges modify the conductance of the WSe_2_ channel. The high responsivity and the excellent reliability indicate that the designed MoS_2_/WSe_2_ p–n heterojunction may achieve ultrasensitive photodetection in the future.

## Conclusions

In this work, we developed a controllable two-step growth method to prepare vertically stacked MoS_2_/WSe_2_ heterostructures. Furthermore, PL Spectroscopy, Raman spectroscopy, and HRTEM were used to characterize the obtained p–n heterojunctions. The results indicate that the samples are of high crystal quality. A p–n field effect transistor was fabricated based on the heterostructures. The bipolar behavior was observed and the device exhibited high carrier mobility of up to 9 cm^2^/(V·s). Under the laser illumination, excellent photodetection properties was obtained with the photoresponsivity approaching 8 A/W and detectivity of 2.93 × 10^11^ Jones. Meanwhile, stable on/off photoswitching was also demonstrated. All the results show that the achieved MoS_2_/WSe_2_ p–n heterojunctions have great potential in integrated optoelectronic applications.

## Supplementary Information

Below is the link to the electronic supplementary material.

**Additional file 1**: **Fig. S1**. Schematic view of CVD growth of MoS_2_/WSe_2_ vertical heterostructures. **Fig. S2** Atomic force microscopy (AFM) image of the MoS_2_ and the MoS_2_/WSe_2_ heterostructures. (a) AFM image of MoS_2_. (b) AFM image of WSe_2_. (c) AFM image of single WSe_2_ triangle. **Fig. S3**. Chemical states of Mo, S, W and Se in the MoS_2_/WSe_2_ heterostructures, measured by X-ray photoelectron spectroscopy. **Fig. S4**. (a) Photocurrent plots under different values of power and gate voltage (*V*_ds_=1 V). (b) Photoresponsivity of the photodetector at various gate voltages ranging from 0 to 60 V (*V*_ds_=1 V). (c) Detectivity of the photodetector at various gate voltages ranging from 0 to 60 V (*V*_ds_=1 V).
