# Supplementary material for: Van der Waals epitaxial growth and optoelectronics of a vertical MoS2/WSe2 p–n junction
Source: Front Optoelectron. 2022 Oct 11;15(1):41. doi: 10.1007/s12200-022-00041-4 (PMC9756242; doi:10.1007/s12200-022-00041-4)
Supplement: Supplementary file 1 — Additional file 1: Fig. S1. Schematic view of CVD growth of MoS2/WSe2 vertical heterostructures. Fig. S2 Atomic force microscopy (AFM) image of the MoS2 and the MoS2/WSe2 heterostructures. (a) AFM image of MoS2. (b) AFM image of WSe2. (c) AFM image of single WSe2 triangle. Fig. S3. Chemical states of Mo, S, W and Se in the MoS2/WSe2 heterostructures, measured by X-ray photoelectron spectroscopy. Fig. S4. (a) Photocurrent plots under different values of power and gate voltage (Vds=1 V). (b) Photoresponsivity of the photodetector at various gate voltages ranging from 0 to 60 V (Vds=1 V). (c) Detectivity of the photodetector at various gate voltages ranging from 0 to 60 V (Vds=1 V). [file 12200_2022_41_MOESM1_ESM.pdf]

Supporting Information

Van der Waals epitaxial growth and optoelectronics of a vertical MoS_2/WSe_2 p-n junction

Yu XIAO,[1] Junyu QU,[1] Ziyu LUO,[1] Ying CHEN,[1] Xin YANG,[1] Danliang ZHANG,[2] Honglai LI,[1] Biyuan ZHENG,[1] Jiali YI,[1] Rong WU,[1] Wenxia YOU,[1] Bo LIU,[1] Shula CHEN,[1,*] Anlian PAN,[1,*]

1 Key Laboratory for Micro-Nano Physics and Technology of Hunan Province, College of Materials Science and Engineering, Hunan University, Changsha 410082, China

2 School of Materials Science and Engineering, Key Laboratory for Micro-Nano Physics and Technology of Hunan Province, Hunan University, Changsha 410082, China

E-mails: shuch@hnu.edu.cn, anlian.pan@hnu.edu.cn

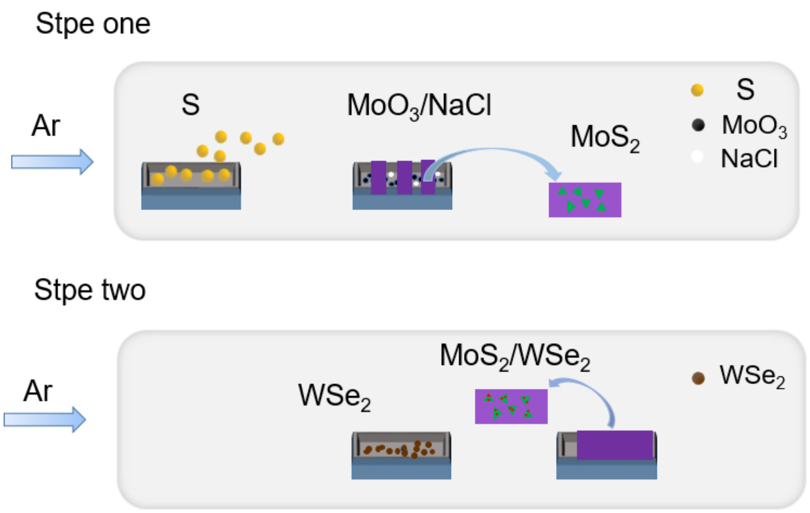

Fig. S1 Schematic view of CVD growth of MoS_2/WSe_2 vertical heterostructures.

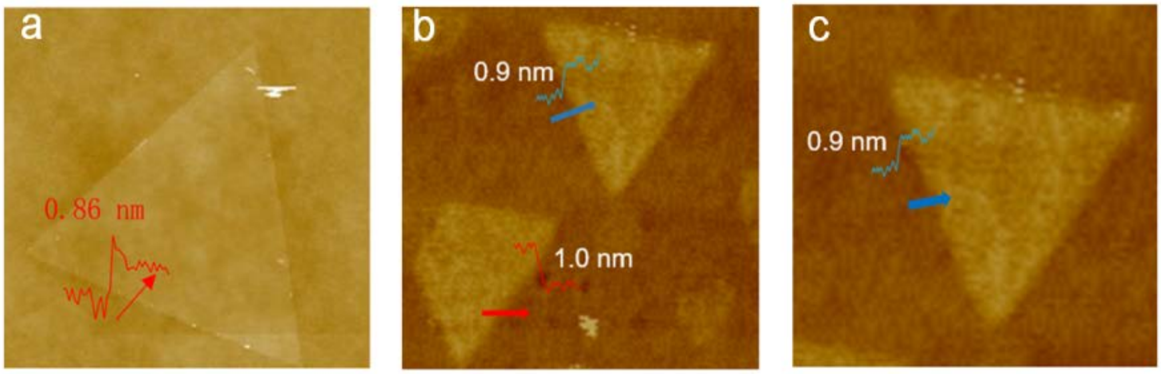

Fig. S2 Atomic force microscopy (AFM) image of the MoS_2 and the MoS_2/WSe_2 heterostructures. (a) AFM image of MoS_2. (b) AFM image of WSe_2. (c) AFM image of single WSe_2 triangle.

Heterojunction transfer method

1. Spin-coat the sample with PMMA on the homogenizer for one minute;
2. Heat the glued sample at 160℃ for 5 min to evaporate the anisole solvent;
3. Cut the four corners of SiO_2/Si;
4. Put it in 2 mol/L KOH and soak at room temperature for about 6 hours. The PMMA film with the material will automatically separate from the SiO_2/Si substrate and float to the surface of the solution;
5. Take out the membrane, clean it in deionized water, and adhere to the prepared copper mesh;
6. Put the copper mesh in an oven at 60 ℃ to dry the deionized water and to make the PMMA film adhere to the copper mesh closely;
7. Remove PMMA in acetone solution, soak for about 5 minutes, and remove the copper mesh. At this time, only the nanosheets are left.

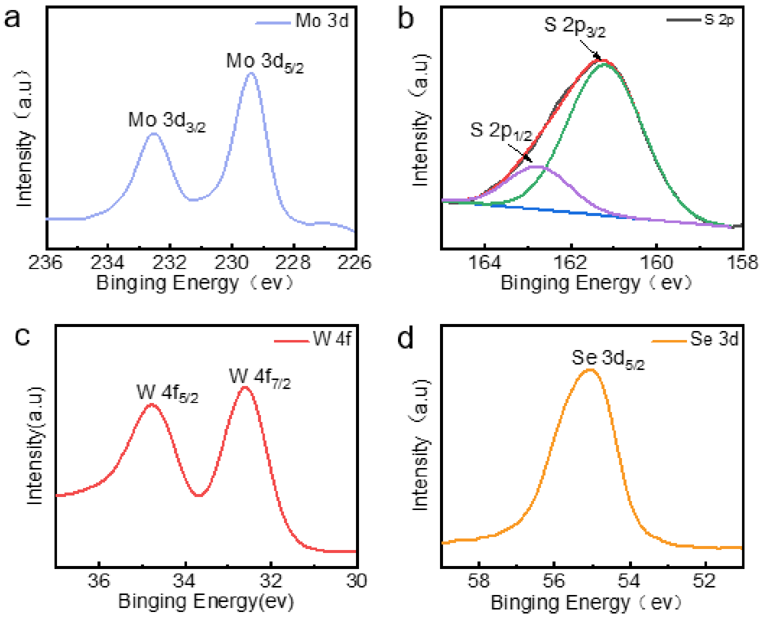

Fig. S3 Chemical states of Mo, S, W and Se in the MoS_2/WSe_2 heterostructures, measured by X-ray photoelectron spectroscopy.

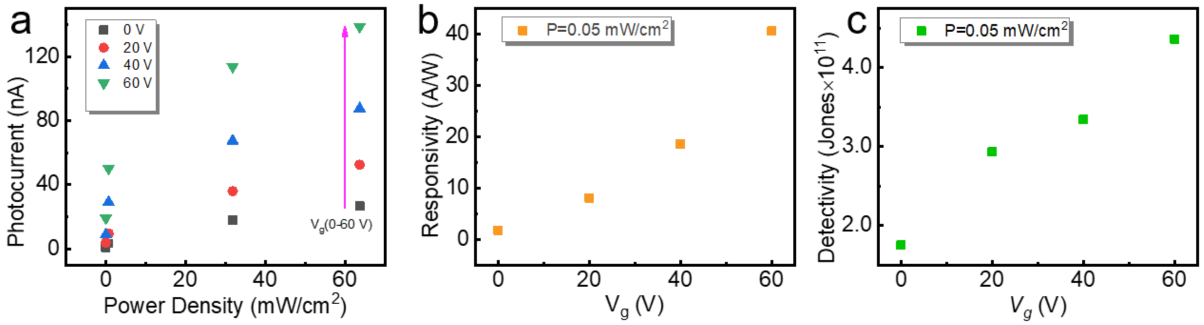

Fig. S4 (a) Photocurrent plots under different values of power and gate voltage (V_{ds}=1 V). (b) Photoresponsivity of the photodetector at various gate voltages ranging from 0 to 60 V (V_{ds}=1 V). (c) Detectivity of the photodetector at various gate voltages ranging from 0 to 60 V (V_{ds}=1 V).
